# *Trans*-(±)-TTPG-B Attenuates Cell Cycle Progression and Inhibits Cell Proliferation on Cholangiocarcinoma Cells

**DOI:** 10.3390/molecules28217342

**Published:** 2023-10-30

**Authors:** Thidarath Rattanaburee, Chompunud Chompunud Na Ayudhya, Tienthong Thongpanchang, Varomyalin Tipmanee, Potchanapond Graidist

**Affiliations:** 1 Department of Biomedical Sciences and Biomedical Engineering, Faculty of Medicine, Prince of Songkla University, Songkhla 90110, Thailand; thidarath.r@rsu.ac.th (T.R.);; 2 Biochemistry Unit, Department of Medical Sciences, Faculty of Science, Rangsit University, Pathum Thani 12000, Thailand; 3 Department of Chemistry and Center of Excellence for Innovation in Chemistry (PERCH-CIC), Faculty of Science, Mahidol University, Bangkok 10400, Thailand; tienthong.tho@mahidol.ac.th

**Keywords:** kusunokinin, *trans*-(±)-TTPG-B, anti-cancer, cholangiocarcinoma

## Abstract

This research aimed to determine the target protein and molecular mechanism of *trans*-(±)-kusunokinin ((±)-KU) derivatives (*trans*-(±)-ARC and *trans*-(±)-TTPG-B). Molecular docking was used to predict potential synthesized (±)-KU targets among 22 proteins. The (±)-TTPG-B bound HSP90α better than EC44, native (±)-KU and (-)-KU, and (±)-KU and (−)-ARC. In contrast, (−)-ARC bound PI3K more strongly than any other test compound. CSF1R and AKR1B1 were not supposed to be the target of (±)-TTPG-B and (±)-ARC, unlike native (±)-KU. The (±)-TTPG-B bound Tyr139 and Trp162 of HSP90α. Moreover, (−)-ARC bound PI3K via hydrogen bonds and π-π stacking at distinct amino acids, which was different from the other tested compounds. Using half of the IC_50_ concentration, (±)-TTPG-B, (±)-KU and (±)-ARC enhanced cell cycle arrest at the G0/G1 phase after 12 h and 24 h on KKU-M213 (CCA) cells. The (±)-TTPG-B showed a stronger inhibitory effect than (±)-ARC and (±)-KU on HSP90α, PI3K, HSP90β, c-Myc, AKT, MEK1, CyclinB1, CyclinD1, and CDK1 for 24 and 48 h after treatment with the same concentration (0.015 µM). Thus, *trans*-(±)-TTPG-B, a newly synthesized compound, has pharmacological potential for development as a target therapy for CCA treatment.

## 1. Introduction

Cholangiocarcinoma (CCA), has been reported to have a hepatocyte origin [1] and is an adenocarcinoma that begins in the bile ducts. Most CCA patients are diagnosed with an advanced and aggressive malignancy that cannot be treated with surgery alone [2]. Targeted therapy is an interesting treatment for improving overall survival and decreasing the undesirable adverse effects of cancer treatment. This treatment uses drugs that bind to a specific protein in cancer [3]. The proteins in the tyrosine kinase pathway are important to cancer growth; therefore, the proteins in this pathway are good targets for targeted therapy. Fibroblast growth factor receptor 2 (FGFR2) is one of the important targets in CCA, and it is targeted by pemigatinib. This drug received approval by the United States Food and Drug Administration for the treatment of adults with unresectable, locally advanced, or metastatic CCA in 2020 [4]. Zanidatamab, a human epidermal growth factor receptor 2 (HER2)-targeted bispecific antibody, is suitable for advanced, unresectable, and metastatic HER2-expressing biliary tract cancers [3]. Activation of the phosphoinositide-3-kinase (PI3K)/AKT signaling pathway is frequently found in CCA [5]. It has been suggested that it is a key step in cancer chemotherapy resistance, especially during DNA-damaging agent treatments such as cisplatin and oxaliplatin [6]. Binimetinib (an MEK1/2 inhibitor) is used in combination with capecitabine. This drug demonstrates promising antitumor efficacy for CCA patients whose first-line chemotherapy treatments failed, especially in patients with RAS/RAF/MEK/ERK pathway mutations [7].

Synthetic (±)-KU has cytotoxic activity on many cancer cells, including breast, CCA, colon, and ovarian cancer. This racemic compound inhibits topoisomerase II, STAT3, CyclinD1, and p21 in breast cancer and CCA [8]. The (−)-KU from black pepper (*Piper nigrum*) decreases tumor size and tumor-related proteins in breast cancer rats [9]; (−)-KU binds CSF1R (computational prediction), a tyrosine kinase family protein, leading to the reduction of cancer proteins such as CSF1R, AKT, CyclinD1, and CDK in breast cancer cells. However, (−)-KU still has a different action on cancer-related proteins when compared with pexidartinib (a potent CSF1R inhibitor) [10]. In addition, (−)-KU also binds MMP-12, HSP90-α, CyclinB1, MEK1 [10], and AKR1B1 [11]. The binding of (−)-KU with AKR1B1 causes downregulation of AKR1B1 and its downstream proteins (PKC, NF-kB, AKT, Nrf2, COX2, and Twist2) in ovarian cancer cells. (−)-KU inhibits aldose reductase activity; however, its activity is weaker than epalrestat (a potent AKR1B1 inhibitor) [12].

**Figure 1:**
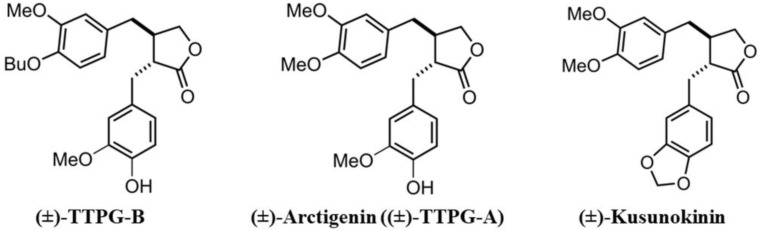
*Trans*-(±)-TTPG-B, *trans*-(±)-arctigenin ((±)-TTPG-A), and *trans-*(±)-kusunokinin.

Previously, we synthesized two *trans*-(±)-KU derivatives, namely *trans*-(±)-TTPG-A (or arctigenin (ARC)) and *trans*-(±)-TTPG-B (TTPG-B) (Figure 1). Interestingly, (±)-TTPG-B showed a stronger cytotoxicity to CCA cells (KKU-M213) (IC_50_ at 0.01 ± 0.001 µM) than (±)-KU (4.47 ± 0.04 µM) and (±)-ARC (0.07 ± 0.01 µM). Moreover, (±)-TTPG-B and (±)-ARC exhibited less toxicity than native (±)-KU on L-929 cells (normal fibroblasts) [13]. 

Due to the different actions of these (±)-KU derivatives, a study on the mechanism of action of (±)-TTPG-B and (±)-ARC on CCA was valuable. First, (±)-ARC and (±)-TTPG-B were predicted target proteins compared with (±)-KU using molecular docking. Then, cell cycle arrest was performed followed by the determination of target proteins and their downstream proteins using Western blotting.

## 2. Results

### 2.1. Molecular Docking of (±)-TTPG-B and (±)-ARC

Both (±)-TTPG-B and (±)-ARC were recently reported to have anticancer properties [13]. To predict the target protein of both (±)-KU compounds, molecular docking was employed similarly to previous studies [10,11]. The binding location and interactions were visualized in order to investigate the binding characteristics. Among the 22 selected proteins were the target protein of native (±)-KU and cancer-related proteins, Table 1. The binding energy (ΔG_bind_) of the selected synthesized compounds ((−)-ARC, (+)-ARC, (−)-TTPG-B, and (+)-TTPG-B) was lower than that of native (±)-KU and a known inhibitor of the target protein. 

The docking results were validated by performing a redocking experiment with the known inhibitor and its protein target. In comparison to the corresponding crystal structure, the criteria were based on the position of the docked pose and its binding pocket site. In the absence of the co-crystallized inhibitor, the docked position of the known inhibitor must resemble the reported binding site. All of the docked inhibitors occupied the same binding site as the experimental structure or literature-reported site.

As CSF1R was reported to be a target of *trans*-(±)-kusunokinin [10], (−)-ARC (−11.41 kcal/mol) showed lower ΔG_bind_ than (+)-KU (−10.58 kcal/mol). The ΔG_bind_ of (−)-TTPG-B (−8.96 kcal/mol) and (+)-TTPG-B (−8.96 kcal/mol) were higher than (−)-KU (−12.01 kcal/mol) and (+)-KU (−10.58 kcal/mol). Therefore, (−)-TTPG-B and (+)-TTPG-B exhibited less tendency to bind CSF1R. (−)-TTPG-B and (+)-TTPG-B may have differential possible target proteins from (−)-KU and (+)-KU. 

The possible target proteins of (+)-TTPG-B were thus HSP90α and EGFR. Considering the previously reported CSF1R and AKR1B1 as the native (±)-KU target, both synthesized (±)-TTPG-B isomers showed higher ΔG_bind_ with CSF1R but similar ΔG_bind_ with AKR1B1. This implied that the synthesized trans-TTPG-B could act on another target, not only CSF1R and AKR1B1. 

The highest ΔG_bind_ with PI3K was from (−)-ARC (−9.77 kcal/mol), compared with (+)-ARC (−9.13 kcal/mol), (−)-TTPG-B (−8.17 kcal/mol), (+)-TTPG-B (−8.77 kcal/mol), (−)-KU (−9.62 kcal/mol), (+)-KU (−8.89 kcal/mol), and apitolisib (−9.54 kcal/mol). Since increasing HSP90α and PI3K levels were crucial to the progression of CCA [14,15], HSP90 and PI3K were chosen to be observed and analyzed in detail in the subsequent experiments.

### 2.2. Binding Position of (±)-TTPG-B and (±)-ARC and on HSP90α

**Figure 2:**
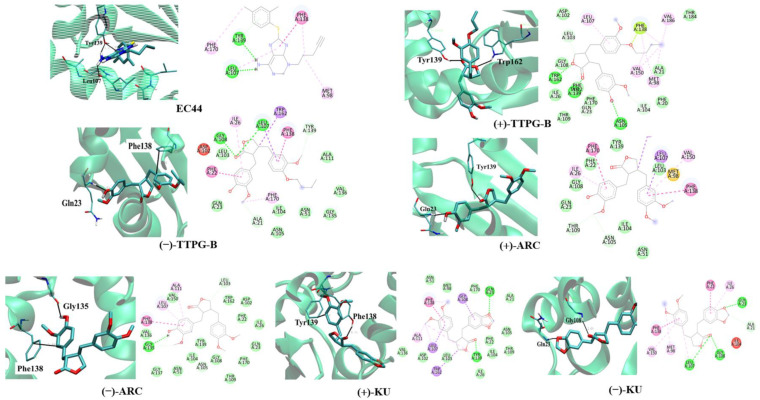
**Ligand interaction with HSP90α structure.** Interactions of the HSP90α structure and its binding domain with EC44 (inhibitor), (−)-TTPG-B; *trans*-(−)-TTPG-B, (+)-TTPG-B; *trans*-(+)-TTPG-B, (+)-ARC; *trans*-(+)-arctigenin, (−)-ARC; *trans*-(−)-arctigenin, (+)-KU; *trans*-(+)-kusunokinin and (+)-KU; *trans*-(+)-kusunokinin.

The binding site and binding interaction(s) between the compound and HSP90α were observed and are concluded in Table 2 and Figure 2. EC44, a known inhibitor of HSP90α, interacted with Tyr139 and Leu107 via π-π stacking and hydrogen bonding, respectively (Figure 2). The native (+)-KU bound to HSP90α via hydrogen bonds at Gly108 and Gln23. Figure 2 demonstrates that native (−)-KU interacted with HSP90α via π-π stacking at Phe138 and Tyr139. The (+)-ARC bound HSP90α with Gln23 and Tyr139 through hydrogen bonds and π-π stacking, whereas (−)-ARC bound HSP90α via Gly135 and Phe138. 

(+)-TTPG-B interacted with HSP90α Tyr139 and Trp162 via π-π stacking. Moreover, (−)-TTPG-B also held the π-π stacking with Phe138 and the hydrogen bond with Gln23. Tyr139 is an important residue of the compound binding to HSP90α. Tyr139 mutually found residue in EC44, (+)-TTPG-B, native (+)-KU, and (+)-ARC. These key amino acids and the low binding energy of HSP90α suggest that (+)-TTPG-B is capable of binding with HSP90α. 

### 2.3. Binding Position of (±)-TTPG-B and (±)-ARC on PI3K

**Figure 3:**
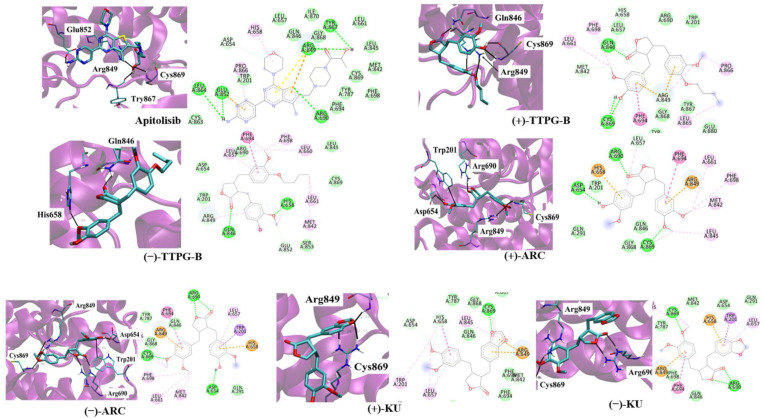
**Ligand interaction with PI3K structure.** Interactions of the PI3K structure and its binding domain with EC44 (inhibitor), (−)-TTPG-B; *trans*-(−)-TTPG-B, (+)-TTPG-B; *trans*-(+)-TTPG-B, (+)-ARC; *trans*-(+)-arctigenin, (−)-ARC; *trans*-(−)-arctigenin, (+)-KU; *trans*-(+)-kusunokinin and (+)-KU; *trans*-(+)-kusunokinin.

Table 3 and Figure 3 depict the interaction between a compound and an amino acid, determining the binding features of PI3K. Apitolisib is a known inhibitor of PI3K, holding Glu852, Arg849, Cys869, and Tyr867. (+)-KU and (−)-KU both possess a hydrogen bond between Arg849 and Cys869 on PI3K. Arg690 additionally interacted with (−)-KU. 

(−)-ARC and (+)-ARC bound PI3K via hydrogen bonding with Cys869, Arg849, Arg649, and Asp654, whereas Trp201 utilized π-π stacking. As shown in Figure 3, (+)-ARC formed hydrogen bonds with Gln846, Cys869, and Arg849. Gln846 and His658 were also bound to (−)-TTPG-B. These results predicted that Trp201 and Cys869 were the essential amino acids on the PI3K active site. (−)-ARC bound to the essential amino acid on PI3K with the lowest binding energy, which was −9.77 kcal/mol. The binding energy of (−)-KU to Cys869 was −9.62 kcal/mol greater than that of (−)-ARC. This result indicates that (−)-ARC is capable of binding with PI3K.

### 2.4. (±)-TTPG-B and (±)-ARC Dominated Cell-Cycle Arrest

**Figure 4:**
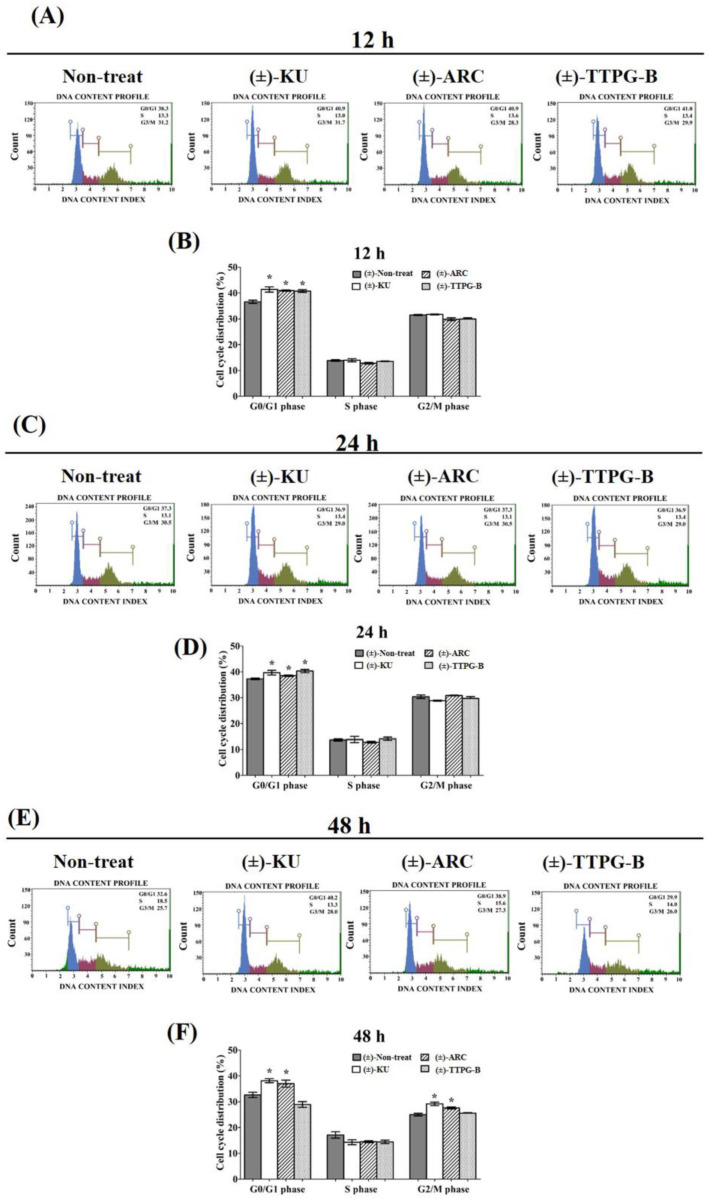
The increasing of the cell cycle arrest by native (±)-KU and its derivatives. KKU-M213 cells were treated with (±)-KU, (±)-ARC, or (±)-TTPG-B at 2.24, 0.035, and 0.005 µM for (**A**,**B**) 12 h, (**C**,**D**) 24 h, and (**E**,**F**) 48 h. Non-treated cells were exhibited as a negative control. Flow cytometry was used for the determination of cell cycle distribution after staining with PI. DNA histograms displayed the G0/G1, S, and G2/M phases. The G0 phase is the resting phase during which the cell has stopped dividing. From the end of the previous M phase to the beginning of DNA synthesis is the G1 phase. All of the chromosomes have been replicated in the S phase. The G2 phase is a period of protein synthesis and rapid cell growth to prepare the cell for mitosis. The distributions of cells phases are exhibited in terms of percentage of mean ± SD (*n* = 3). The asterisk (*) is represents *p* < 0.05 when compared with the non-treated cells (control). (±)-KU; *trans*-(±)-kusunokinin, (±)-ARC; (±)-arctigenin, (±)-TTPG-B; *trans*-(±)-TTPG-B.

In order to determine the inhibitory effect of (±)-KU and its derivatives on cell cycle arrest, KKU-M213 cells were treated with half of IC_50_ for each compound. The results show that (±)-KU (41.40 ± 2.01%), (±)-TTPG-B (40.76 ± 1.07%), and (±)-ARC (40.94 ± 0.46%) were significantly arrested at the G0/G1 phase compared with the non-treated cells (negative control) (36.60 ± 1.28%) at 12 h (Figure 4A,B). In addition, (±)-TTPG-B (41.35 ± 1.23%) showed the highest DNA content at the G0/G1 phase compared with (±)-KU (39.70 ± 1.77%), (±)-ARC (38.52 ± 0.59%), and the non-treated cells (37.30 ± 0.56%) for 24 h after treatment (Figure 4C,D). Interestingly, (±)-KU and (±)-ARC induced cell cycle arrest at G0/G1 and G2/M at 48 h. Meanwhile, only (±)-TTPG-B showed a DNA content lower than (±)-KU, (±)-ARC, and the non-treated cells (Figure 4E,F). These data suggest that the anti-proliferation effect of (±)-TTPG-B on cell cycle arrest is not similar to (±)-KU and (±)-ARC. Therefore, the molecular mechanism of (±)-TTPG-B and (±)-ARC were further evaluated using Western blot analysis.

### 2.5. (±)-TTPG-B and (±)-ARC Suppressed HSP90α and PI3K, and Their Downstream Proteins

**Figure 5:**
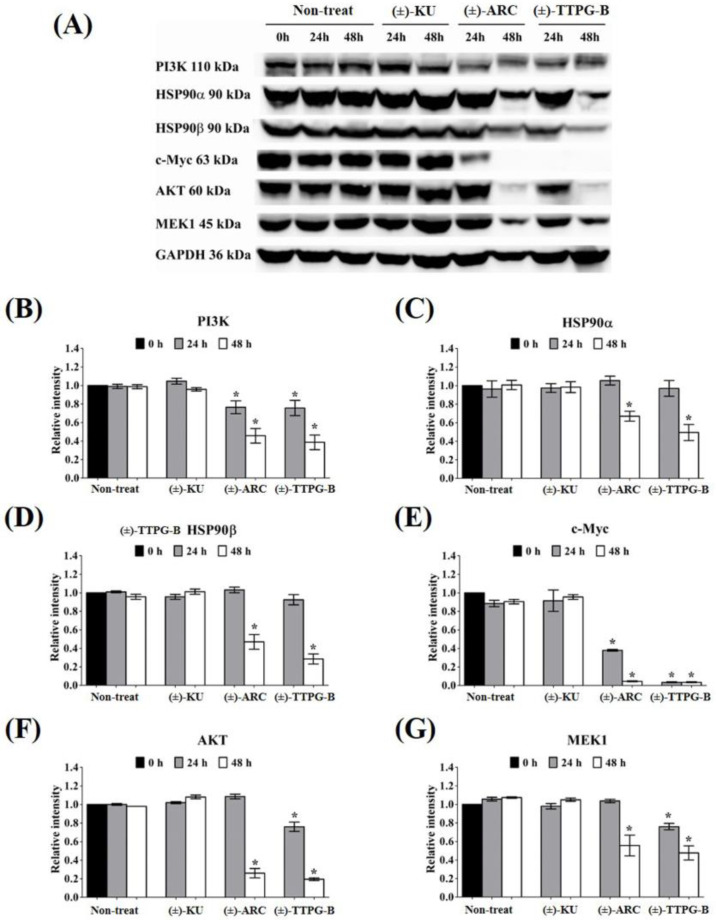
Effect of native *trans*-(±)-KU and its derivatives on HSP9α PI3K and their downstream proteins. KKU-M213 cells were treated with (±)-KU, *trans*-(±)-TTPG-B, or *trans*-(±)-ARC at 0.015 µM for 24 and 48 h. Non-treated cells served as negative controls. (**A**) After incubation, all cells were harvested and lysed by RIPA buffer. The 80 mg of protein was subjected to SDS-PAGE, and interesting proteins were determined using Western blot analysis. GAPDH was used as an internal control. Proteins of interest, including (**B**) PI3K, (**C**) HSP90α, (**D**) HSP90β, (**E**) c-Myc, (**F**) AKT, and (**G**) MEK1, were quantitatively calculated by normalizing the GAPDH band intensities. Data are representative of three independent experiments. All graphs are shown as mean ± SD. Student’s *t*-test was used for consideration of the *p*-value. * *p* < 0.05 vs. control. (±)-KU; *trans*-(±)-kusunokinin, (±)-ARC; (±)-arctigenin, (±)-TTPG-B; *trans*-(±)-TTPG-B.

In order to indirectly validate the protein target and compare the actions of (±)-TTPG-B, (±)-ARC, and (±)-KU, KKU-M213 cells were incubated with these three compounds at the same concentration (0.015 µM) for 24 and 48 h. As shown in Figure 5, the level of PI3K (a predicted target protein of (±)-ARC) was significantly down-regulated after treatment with (±)-TTPG-B and (±)-ARC for 24 and 48 h when compared with (±)-KU and the non-treated cells. HSP90α, a predicted target protein of (±)-TTPG-B, was suppressed by (±)-TTPG-B and (±)-ARC at 48 h. Moreover, HSP90b was reduced by (±)-TTPG-B and (±)-ARC at 48 h. (±)-TTPG-B suppressed AKT and MEK1 (downstream proteins of HSP90α, HSP90β and PI3K) at 24 and 48 h, whereas (±)-ARC proteins were only suppressed at 48 h. Furthermore, (±)-TTPG-B and (±)-ARC showed the strongest inhibitory effect on c-Myc at 48 h. In contrast, (±)-KU did not affect PI3K, HSP90α, HSP90β, c-Myc, AKT, and MEK1. 

**Figure 6:**
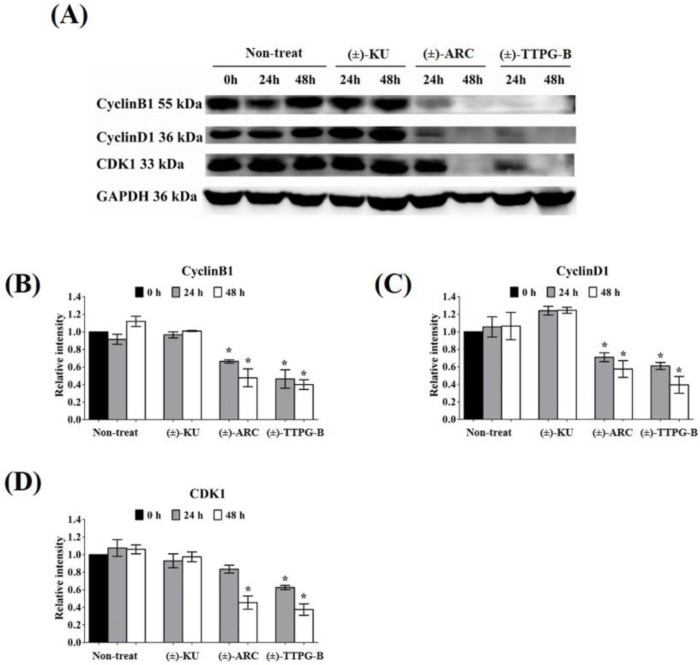
Effect of native (±)-KU and its derivatives on cell proliferation proteins. KKU-M213 cells were treated with native (±)-KU, (±)-TTPG-B, or (±)-ARC at 0.015 µM and incubated for 24 and 48 h. Non-treated cells served as negative controls. (**A**) After incubation, all cells were lysed, and 80 mg of protein was subjected to SDS-PAGE followed by Western blot analysis. Proteins of interest, including (**B**) Cyclin B1, (**C**) Cyclin D1, and (**D**) CDK1, were quantitatively calculated by normalizing the GAPDH band intensities. Data are representative of three independent experiments. All graphs are shown as mean ± SD. * *p* < 0.05, compared with non-treated cells using Student’s *t*-test. (±)-KU; *trans*-(±)-kusunokinin, (±)-ARC; (±)-arctigenin, (±)-TTPG-B; *trans*-(±)-TTPG-B.

Both (±)-TTPG-B and (±)-ARC also suppressed CyclinB1 and CyclinD1, a specific protein in cell proliferation, after 48 h of the treatment. CDK1 also was reduced when treated with (±)-TTPG-B and (±)-ARC. Accordingly, there were no significant decreases in the level of CyclinB1, CyclinD1, and CDK during incubation with native (±)-KU compared with the non-treated cells (Figure 6). 

## 3. Discussion

(±)-KU has an anti-cancer effect and could bind with CSF1R [10] and AKR1B1 [12]. In our previous study, we showed that (±)-TTPG-A (or (±)-ARC) and (±)-TTPG-B were (±)-KU derivative compounds that were modified at two binding positions, including 3,4 dimethoxybenzyl butyrolactone and 1,3-benzodioxole parts. Both derivative compounds showed cytotoxicity in breast cancer, CCA, colon, and ovarian cancer cells. (±)-TTPG-B had a greater cytotoxic and apoptotic induction effect than (±)-KU. In addition, (±)-ARC induced cell cycle arrest at the S phase, whereas (±)-TTPG-B caused cell arrest at the G0/G1 phase, which is the same as (±)-KU in KKU-M213 cells [13]. In contrast, in this study, half of the IC_50_ values of (±)-TTPG-B, (±)-ARC, and (±)-KU caused cell cycle arrest at G0/G1 at 12 and 24 h. Therefore, we hypothesized that the specific target protein of these derivatives might be a protein associated with cell signaling and cell proliferation, which remains similar to (±)-KU. Here, we used the molecular docking technique to determine the binding affinity of these two compounds with twenty-two target proteins of the native (±)-KU.

The results showed that (±)-TTPG-B and (±)-ARC in racemic forms exhibited a lower ability to bind to CSF1R and AKR1B1 than (±)-KU. (±)-TTPG-B showed the highest binding affinity with HSP90α and EGFR; meanwhile, (±)-ARC bound with PI3K. 

**Figure 7:**
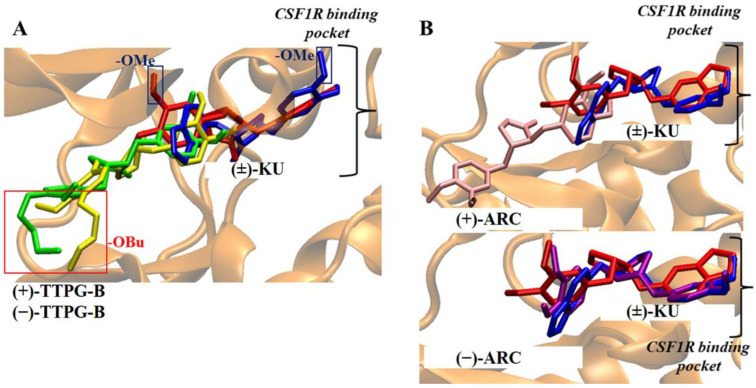
Comparison of *trans*-(±)-TTPG-B and (±)-ARC with (±)-KU on CSF1R binding pocket. (**A**) The red and blue rectangles denote a longer butoxy chain (-OBu) and methoxy (-OMe) in the structure. The red and blue structures depict (+)-KU and (−)-KU, respectively. The green and yellow structures depict (+)-TTPG-B and (−)-TTPG-B, respectively. The black bracket illustrates the drug binding site of CSF1R. The presence of the longer -OBu chain hindered the (±)-TTPG-B from accessing the deep binding pocket, in contrast to the -OMe group in native kusunokinin. (**B**) The red and blue structures depict (+)-KU and (−)-KU, respectively. The pink and purple structures depict (+)-ARC and (−)-ARC, respectively. The black bracket illustrates the drug binding site of CSF1R. The different stereo-configurations between (±)-ARC could be the key to the different binding poses in the CSF1R pocket. (−)-KU; *trans*-(−)-kusunokinin, (+)-KU; *trans*-(+)-kusunokinin, (−)-ARC; *trans*-(−)-arctigenin, (+)-ARC; *trans*-(+)-arctigenin, (−)-TTPG-B; *trans*-(−)-TTPG-B, (+)-TTPG-B; *trans*-(+)-TTPG-B, (±)-KU; *trans*-(±)-kusunokinin, (±)-ARC; *trans*-(±)-arctigenin, (+)-TTPG-B; *trans*-(+)-TTPG-B, (−)-TTPG-B; *trans*-(−)-TTPG-B.

The (±)-TTPG-B compound showed a lower affinity towards CSF1R than (+)-KU and (−)-KU. The CSF1R binding site is characterized by a narrow cleft [10]. The presence of a long chain of butoxy groups (-OBu) introduces steric hindrance to the amino acid at the binding site, in contrast to the smaller methoxy groups (-OMe) found in (+)-KU and (−)-KU. The observation of this feature, resulting from the longer hydrocarbon chain, was made by comparing the poses of (±)-TTPG-B with the (+)-KU poses at the CSF1R pocket, as depicted in Figure 7A. For the (±)-ARC, while the docking suggested that the hydroxy group in both forms could do so, (−)-ARC was the only form resembling the (±)-KU, while the (+)-ARC was not able to penetrate the CSF1R binding site. Thus, we speculated that, in the case of (±)-ARC, the binding with CSF1R of (±)-ARC could be different due to their different stereo-configurations; Figure 7B.

The phenolic group present in (±)-TTPG-B and (±)-ARC may potentially contribute to a decrease in the binding affinity to the hydrophobic pocket of AKR1B1. This is because the primary interactions observed in (±)-KU were π-π stacking and the hydrophobic effect [12], whereas the phenolic group is considered a polar group. Therefore, there was a reduced affinity observed between (±)-TTPG-B and (±)-ARC with AKR1B1. 

Next, the pose position analysis of (±)-TTPG-B and (±)-ARC. The structural information of the ATP binding pocket in the N-terminal domain of HSP90 demonstrated that hydrophobic substituents at the 6 position could reach the side chains of Phe138, Try139, and Trp162 residues and form π-π interactions. (+)-TTPG-B bound stronger to HSP90α than EC44 (a known inhibitor), (±)-KU, and (±)-ARC. (+)-TTPG-B requires Tyr139 and Trp162 via π-π stacking on HSP90α. A new series of compounds bearing 2-thioquinazolinone scaffolds were designed and synthesized as the HSP90 inhibitors. Compound **8a** showed a dual mode of interactions, basically by the same type of binding forces (arenehydrogen and hydrogen bonding), but this time with even more amino acid residues (four residues named Asn51, Tyr139, Val136, and Gly135), which could be in part responsible for its higher activity on HSP90α [16]. The binding positions of (+)-TTPG-B and compound **8a** shared the same key amino acid as Tyr139. Therefore, (+)-TTPG-B might have the capacity to bind to HSP90α.

(−)-ARC had the highest affinity bound on the PI3K protein (−9.77 kcal/mol). (−)-ARC and (+)-ARC interfered with Cys869, Arg849, Arg649, on PI3K residues. Van der Waals forces created around the NSC777213 as a PI3K inhibitor backbone with the Pro789, Tro292, Glu880, Cys869, Gln846, and Ile870 residues of PI3K [17,18]. The trp201, Leu657, Gln846, and Phe698 residues of PI3K p110 formed a hydrophobic pocket, and evodiamine (EVO) bound to this active pocket [19].

The main forces between EVO and the residues were the Van der Waals forces, π-π stacking, and hydrophobic interactions. The docking results of evodiamine amino derivative (EVA) and PI3K p110 suggested that EVO was also bound in the same hydrophobic pocket composed of the above amino acid residues, and there was a hydrogen bond between Cys869 and the amino group at the C10 position of EVA [19]. (−)-ARC and NSC777213 bound to Cys869. Therefore, (−)-ARC showed the ability to act as a PI3K inhibitor. The molecular docking results predicted that (±)-TTPG-B, (±)-ARC, and (±)-KU displayed different specific target proteins. In addition, the long butoxy chain in (±)-TTPG-B could be responsible for more affinity towards HSP90a and PI3K than (±)-ARC.

**Figure 8:**
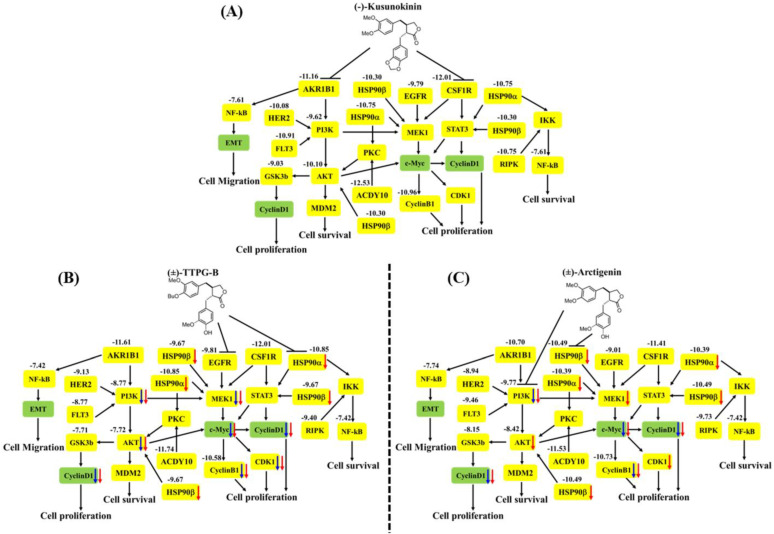
The proposed pathway of (**A**) native *trans*-(−)-kusunokinin, (**B**) *trans*-(±)-TTPG-B, and (**C**) *trans*-(±)-arctigenin on CCA cells. The bold black arrows refer to the inhibitory effect of the compounds. The numbers represent the binding energy in kcal/mol, and the yellow box refers to the proteins from the molecular docking analysis. The blue and red arrows mention the reduction of the protein levels in the KKU-M213 cells at 24 and 48 h, respectively, using Western blot analysis.

In order to indirectly verify the results from the molecular docking that (±)-TTPG-B and (±)-ARC bound with HSP90α and PI3K, respectively, in contrast with (±)-KU, the related proteins of HSP90α and PI3K were detected. We found that 0.015 µM (±)-KU did not affect all the tested proteins (PI3K, HSP90α, HSP90β, c-Myc, AKT, MEK1, CyclinB1, CyclinD1, and CDK1), which could be due to the low concentration (Figure 8A). Interestingly, 0.015 µM (±)-TTPG-B strongly affected its target protein (HSP90α and EGFR) and downstream with the related cell proliferation pathway (Figure 8B). Surprisingly, treatment of KKU-M213 cells with a concentration 4.67 µM-fold lower than the IC_50_ of (±)-ARC showed strong suppression of its target proteins, including HSP90α and PI3K, with regulated cell proliferation (Figure 8C).

Target proteins for CCA treatment have been reported, such as FGFR, IDH1, HER2, NTRK, PI3K, and MAPK [3,20]. The drugs in a clinical study for targeted therapies with FDA approval, including pemigatinib, ivosidenib, zanidatamab, and futibatinib, which target FGFR, IDH1, HER2, and FGFR, respectively [3]. The PI3K-AKT and RAS-MAPK pathways are promising pathways for targeted therapy for CCA due to this pathway being related to cell proliferation, angiogenesis, and survival. Tyrosine kinase proteins of both receptor and non-receptor types are also target proteins, including HER2, EGFR, VEGFR, PDGFR, and FGFR. These proteins are the upstream PI3K-AKT and RAS-MAPK pathways [19]. HSP90 is an ATP-dependent molecular chaperon highly expressed in many cancer types [21]. HSP90 controls many proteins in cancer which play an essential role in cell growth and survival; hence, the development of HSP90 inhibitors can be beneficial in cancers that have a high HSP90 expression (Figure 8) [22]. Taken together, (±)-TTPG-B could be a drug target for CCA-targeted therapy. However, the anti-cancer activity and side effects of this compound require more in-depth studies in the animal model. 

## 4. Materials and Methods

### 4.1. Compound Acquisition

*Trans*-(±)-KU was synthesized following the procedure reported in our previous publication [8]. The synthetic pathways of *trans*-(±)-ARC and *trans*-(±)-TTPG-B were also exhibited in our previous publication [10].

### 4.2. Molecular Docking

Twenty-two proteins associated with CCA cells and a potential target protein of native (±)-KU were selected for the investigation of potential protein targets of (±)-ARC and (±)-TTPG-B. The 3D protein structures were obtained from the Research Collaboratory for Structural Bioinformatics Protein Database Protein Data Bank (RCSB PDB). The AutoDockTool (ADT) version 4.1 was used to separate water molecules and all co-crystallized ligands. Every co-crystallized ligand was eliminated. All polar hydrogen atoms were included in the Protein Data Bank (PDB) file format in order to simulate hydrogen bond interactions. The RCSB PDB ascension codes of the 22 selected proteins are concluded in the Supplementary Information (Table S1).

The conformers, as the SDF format file, of native (+)-KU, native (−)-KU, (+)-ARC, (−)-ARC, (+)-TTPG-B, (−)-TTPG-B, and known ligands were obtained from the PubChem database. All structure files were then converted to the PDB file format using the Online SMILES Translator and Structure File Generator (https://cactus.nci.nih.gov/translate/, accessed on 21 January 2022). ADT was used to add all of the missing polar hydrogen atoms. Finally, both protein and ligand structures were saved in a PDB and Partial Charge (Q) and Atom Type (T) (PDBQT) file. 

A molecular docking study between all compounds and the 22 selected proteins was evaluated using AutoDock4 version 4.2 [10]. The grid was positioned at the center of the protein molecule with an x–y–z grid point of 126–126–126 cubic angstrom (Å^3^). The Fifty Lamarckian Genetics Algorithm (GA), which runs with a population size of 200, was used in the study. All other parameters were placed at the default value on the AutoDock4 program. 

The docking score was reported as the lowest predicted binding energy (ΔG_bind_) in kcal/mol. The docking outcomes were validated by performing a redocking experiment with the known inhibitor and its protein target. The criteria were based on the position of the docked pose and the site of its binding pocket as compared to the corresponding crystal structure. The docked position of the known inhibitor had to resemble the reported binding site when the co-crystallized inhibitor was absent.

The Visual Molecular Dynamics (VMD) package was used for all 3D structure visualizations. Biovia Discovery Studio (DS) was used to analyze the 2D interaction between a compound and a protein based on its PDB file structure.

### 4.3. Cell Culture

Human CCA (KKU-M213) cells were kindly donated by Assistant Professor Dr. Mutita Junking (Division of Molecular Medicine, Department of Research and Development, Faculty of Medicine Siriraj Hospital, Mahidol University, Bangkok, Thailand). Cells were grown in Dulbecco’s Modified Eagle Medium (DMEM) and supplemented with 10% fetal bovine serum (FBS) (Invitrogen, Waltham, MA, USA), L-glutamine (2 µM) (Invitrogen), and an antibiotic mixture of penicillin (100 U/mL) and streptomycin (100 μg/mL) (Invitrogen). Cells were cultured at 37 °C with 5% CO_2_ in a humidified incubator [8].

### 4.4. Cell Cycle Analysis Assay

The assay was performed using the Muse^®^ kit (Merck Millipore, Darmstadt, Germany) according to the manufacturer’s protocol. In brief, KKU-M213 cells were seeded in 24-well plates at a density of 1 × 10^5^ cells/well. Cells were starved with low fetal bovine serum in DMEM for 24 h. Then, the medium was removed and replaced with a fresh culture medium without or with at 2.24, 0.035, and 0.005 µM of native (±)-KU, (±)-ARC, or (±)-TTPG-B and incubated for 12, 24, and 48 h. Cell pellets were resuspended in 1X PBS and the cell distribution was determined using propidium iodid (PI). The percentage of the cells in the G0/G1, S, and G2/M phases was analyzed using the MUSE^®^ Cell Analyzer (Merck Millipore, Darmstadt, Germany).

### 4.5. Western Blot Analysis

KKU-M213 cells were seeded in 6-well plates with 1 × 10^6^ cells/well and treated with 0.15 µM (±)-KU, (±)-TTPG-B, or (±)-ARC for 24 and 48 h. After treatment, cells were subjected to Western blot analysis, as previously reported [8]. Cells were harvested by trypsinization and lysated using a RIPA buffer (Thermo Scientific, Waltham, MA, USA). According to the manufacturer’s instructions, the total protein concentration was measured using the Bradford method (Bio-Rad, Hercules, CA, USA). Eighty micrograms of each protein lysate were separated on 12% sodium dodecyl sulfate-polyacrylamide gel electrophoresis (SDS-PAGE) and transferred to a nitrocellulose membrane (Millipore, Billerica, MA, USA). After that, the membranes were blocked with 5% non-fat dry milk in TBST (0.1% Tween 20, 154 mM NaCl, 48 mM Tris-base, pH 6.8) to prevent the non-specific binding. The membranes were exposed to primary antibodies, including anti-PI3K, HSP90α, HSP90β, c-Myc, CyclinD1, MEK1, AKT, (Cell Signaling Technology, Danvers, MA, USA), CDK1, CyclinB1 (Santa Cruz Biotechnology, Dallas, TX, USA), and GAPDH antibodies (Calbiochem, Darmstadt, Germany). The protein signal was visualized using the SuperSignal^TM^ West Dura Extended Duration substrate kit (Thermo Scientific, Waltham, MA, USA), according to the protocol supplied with the kit and visualized using a CCD camera. The band intensity was analyzed by Image J (NIH, Bethesda, MD, USA).

### 4.6. Statistical Analysis

Data values of 3 independent experiments were analyzed by Student’s *t*-test on Microsoft Excel and were represented as the mean ± standard deviation (SD). A *p*-value of less than 0.05 was considered to indicate a statistically significant difference between groups.

## 5. Conclusions

Molecular docking showed that (±)-TTPG-B and (±)-ARC exhibited a lower binding affinity to CSF1R and AKR1B1 than (−)-KU and (+)-KU, respectively. (+)-TTPG had potential interactions with HSP90α and EGFR with a stronger binding energy than all of the tested compounds, including selected inhibitors. In addition, (−)-ARC showed an interesting binding affinity to PI3K and HSP90β. The racemates of (±)-TTPG-B, (±)-ARC, and (±)-KU enhanced cell cycle arrest at G0/G1 on KKU-M213 cells. Using a similar concentration (0.015 µM) of each compound and incubation at 24 and 48 h, (±)-TTPG-B showed a significantly decreased PI3K, c-Myc, AKT, MEK1, CyclinB1, CyclinD1, and CDK1; meanwhile, (±)-ARC showed a significantly decreased PI3K, c-Myc, CyclinB1, and CyclinD1. Both (±)-TTPG-B and (±)-ARC suppressed HSP90α and HSP90β at 48 h after treatment. In summary, the specific target proteins of (±)-TTPG-B and (±)-ARC may be HSP90α and PI3K or upstream of these proteins with the associated cell proliferation.

## Figures and Tables

**Table 1:** Potential targets of (±)-KU and its derivatives.

No.	Protein	Docking Score (kcal/mol^−1^)							Name
		(−)-KU	(+)-KU	(−)-ARC	(+)-ARC	(−)-TTPG-B	(+)-TTPG-B	Known Inhibitor	
	**Cell signaling**								
1.	ACDY10	−12.53	−11.80	−11.41	−11.53	−11.74	−11.43	−8.23	SQ-22536
2.	AKT	−10.10	−10.26	−8.42	−8.30	−7.72	−7.09	−9.50	Capivasertib
3.	CSF1R	−12.01	−10.58	−11.41	−9.29	−8.96	−9.35	−12.29	Pexidartinib
4.	EGFR	−9.79	−9.55	−9.09	−9.01	−8.71	−9.81	−9.41	Erlotinib
5.	FLT3	−10.91	−9.42	−9.46	−8.53	−8.77	−8.58	−10.56	Gilteritinib
6.	GSK3b	−8.24	−9.03	−7.83	−8.15	−7.71	−7.60	−5.37	Tideglusib
7.	HER2	−9.25	−10.08	−8.94	−8.37	−9.13	−8.96	−9.03	SCHEMBL3505765
8.	Hsp90α	−10.75	−10.52	−10.39	−9.66	−10.12	−10.85	−9.59	EC44
9.	Hsp90β	−10.30	−10.57	−10.49	−8.83	−9.30	−9.67	−10.45	Sunitinib
10.	JAK1	−8.29	−8.46	−8.27	−9.01	−7.98	−7.62	−10.54	CHEMBL3779913
11.	MDM2	−7.32	−7.72	−6.69	−7.78	−7.02	−7.04	−9.11	CHEMBL1234332
12.	MEK1	−10.38	−10.72	−10.10	−8.99	−9.80	−9.40	−13.09	Trametinib
13.	mTOR	−6.91	−7.10	−6.70	−7.11	−6.61	−6.90	−7.48	AZD8055
14.	NF-kB	−7.61	−7.80	−7.74	−7.22	−7.42	−6.88	−7.16	BAY11-7085
15.	PI3K	−9.62	−8.89	−9.77	−9.13	−8.17	−8.77	−9.54	Apitolisib
16.	PKC	−9.89	−9.74	−9.01	−8.36	−8.65	−8.27	−13.33	Sotrastaurin
17.	RIPK	−10.41	−10.75	−9.73	−9.89	−9.37	−9.40	−9.00	SCHEMBL4397912
18.	STAT3	−7.68	−7.51	−7.31	−7.20	−6.66	−6.94	−9.90	STAT5-IN-2
	**Cell invasion**								
1.	AKR1B1	−11.16	−11.80	−10.60	−10.70	−11.61	−11.40	−10.18	Epalrestat
2.	VEGFR	−8.93	−9.53	−9.10	−9.48	−8.72	−8.66	−11.93	Tivazanib
	**Cell cycle**								
1.	CDK1	−10.42	−10.06	−10.06	−9.51	−10.15	−9.63	−11.32	CGP74514A
2.	CyclinB1	−10.96	−10.41	−10.52	−10.73	−10.58	−10.35	−9.40	Q27097368

Note: (−)-KU; *trans*-(−)-kusunokinin, (+)-KU; *trans*-(+)-kusunokinin, (−)-ARC; *trans*-(−)-arctigenin, (+)-ARC; *trans*-(+)-arctigenin, (−)-TTPG-B; *trans*-(−)-TTPG-B, (+)-TTPG-B; *trans*-(+)-TTPG-B.

**Table 2:** Interacting amino acids of HSP90α with the tested compounds.

HSP90α						
EC44 (Inhibitor)	(+)-TTPG-B	(−)-TTPG-B	(+)-ARC	(−)-ARC	(+)-KU	(−)-KU
Leu107		Leu107				Leu107
Tyr139	Tyr139		Tyr139		Tyr139	
		Gln23	Gln23			Gln23
		Phe138		Phe138	Phe138	
	Trp162	Gly108		Gly135		Gly108

The highlighted amino acids indicate those that interacted with the inhibitor.

**Table 3:** Interacting amino acids of PI3K with the tested compounds.

PI3K						
Apitolisib (Inhibitor)	(+)-TTPG-B	(−)-TTPG-B	(+)-ARC	(−)-ARC	(+)-KU	(−)-KU
Arg849	Arg849		Arg849	Arg849	Arg849	Arg849
Gln852						
Tyr867						
Cys869	Cys869		Cys869	Cys869	Cys869	Cys869
			Trp201	Trp201		
			Arg649	Arg649		
			Arg690	Arg690		Arg690
		Gln846		Gln846		
		His658		Asp654		

The highlighted amino acids indicate those that interacted with the inhibitor.

## Data Availability

All data represented in this study are available from the corresponding author upon request.

## Supplementary Materials

The following supporting information can be downloaded at: https://www.mdpi.com/article/10.3390/molecules28217342/s1. Table S1: Name and RCSB code of 22 selected targets of (±)-ARC and (±)-TTPG-B.

## References

[1] Wardell C.P., Fujita M., Yamada T., Simbolo M., Fassan M., Karlic R., Polak P., Kim J., Hatanaka Y., Maejima K. (2018). Genomic characterization of biliary tract cancers identifies driver genes and predisposing mutations. J. Hepatol..

[2] Simile M.M., Bagella P., Vidili G., Spanu A., Manetti R., Seddaiu M.A., Babudieri S., Madeddu G., Serra P.A., Altana M. (2019). Targeted Therapies in Cholangiocarcinoma: Emerging Evidence from Clinical Trials. Medicina.

[3] Cho S.M., Esmail A., Raza A., Dacha S., Abdelrahim M. (2022). Timeline of FDA-Approved Targeted Therapy for Cholangiocarcinoma. Cancers.

[4] Hoy S.M. (2020). Pemigatinib: First Approval. Drugs.

[5] Fouassier L., Marzioni M., Afonso M.B., Dooley S., Gaston K., Giannelli G., Rodrigues C.M.P., Lozano E., Mancarella S., Segatto O. (2019). Signalling networks in cholangiocarcinoma: Molecular pathogenesis, targeted therapies and drug resistance. Liver Int. Off. J. Int. Assoc. Study Liver.

[6] Wang L., Zhao X., Fu J., Xu W., Yuan J. (2021). The Role of Tumour Metabolism in Cisplatin Resistance. Front. Mol. Biosci..

[7] Kim J.W., Lee K.H., Kim J.W., Suh K.J., Nam A.R., Bang J.H., Bang Y.J., Oh D.Y. (2019). Enhanced antitumor effect of binimetinib in combination with capecitabine for biliary tract cancer patients with mutations in the RAS/RAF/MEK/ERK pathway: Phase Ib study. Br. J. Cancer.

[8] Rattanaburee T., Thongpanchang T., Wongma K., Tedasen A., Sukpondma Y., Graidist P. (2019). Anticancer activity of synthetic (±)-kusunokinin and its derivative (±)-bursehernin on human cancer cell lines. Biomed. Pharmacother. Biomed. Pharmacother..

[9] Tedasen A., Dokduang S., Sukpondma Y., Lailerd N., Madla S., Sriwiriyajan S., Rattanaburee T., Tipmanee V., Graidist P. (2020). (-)-Kusunokinin inhibits breast cancer in N-nitrosomethylurea-induced mammary tumor rats. Eur. J. Pharmacol..

[10] Rattanaburee T., Tipmanee V., Tedasen A., Thongpanchang T., Graidist P. (2020). Inhibition of CSF1R and AKT by (±)-kusunokinin hinders breast cancer cell proliferation. Biomed. Pharmacother. Biomed. Pharmacother..

[11] Tanawattanasuntorn T., Thongpanchang T., Rungrotmongkol T., Hanpaibool C., Graidist P., Tipmanee V. (2021). (-)-Kusunokinin as a Potential Aldose Reductase Inhibitor: Equivalency Observed via AKR1B1 Dynamics Simulation. ACS Omega.

[12] Tanawattanasuntorn T., Rattanaburee T., Thongpanchang T., Graidist P. (2022). Trans-(±)-Kusunokinin Binding to AKR1B1 Inhibits Oxidative Stress and Proteins Involved in Migration in Aggressive Breast Cancer. Antioxid..

[13] Rattanaburee T., Sermmai P., Tangthana-Umrung K., Thongpanchang T., Graidist P. (2022). Anticancer Activity of (±)-Kusunokinin Derivatives towards Cholangiocarcinoma Cells. Molecules.

[14] Basset C.A., Conway de Macario E., Leone L.G., Macario A.J.L., Leone A. (2023). The chaperone system in cancer therapies: Hsp90. J. Mol. Histol..

[15] Zhao Q., Zhu H.P., Xie X., Mao Q., Liu Y.Q., He X.H., Peng C., Jiang Q.L., Huang W. (2020). Novel HSP90-PI3K Dual Inhibitor Suppresses Melanoma Cell Proliferation by Interfering with HSP90-EGFR Interaction and Downstream Signaling Pathways. Int. J. Mol. Sci..

[16] El-Shafey H.W., Gomaa R.M., El-Messery S.M., Goda F.E. (2020). Synthetic approaches, anticancer potential, HSP90 inhibition, multitarget evaluation, molecular modeling and apoptosis mechanistic study of thioquinazolinone skeleton: Promising antibreast cancer agent. Bioorganic Chem..

[17] Lawal B., Lo W.C., Mokgautsi N., Sumitra M.R., Khedkar H., Wu A.T., Huang H.S. (2021). A preclinical report of a cobimetinib-inspired novel anticancer small-molecule scaffold of isoflavones, NSC777213, for targeting PI3K/AKT/mTOR/MEK in multiple cancers. Am. J. Cancer Res..

[18] Arthur D.E., Uzairu A. (2019). Molecular docking studies on the interaction of NCI anticancer analogues with human Phosphatidylinositol 4,5-bisphosphate 3-kinase catalytic subunit. J. King Saud. Univ. Sci..

[19] Wang T., Qi D., Hu X., Li N., Zhang X., Liu H., Zhong C., Zhang J. (2021). A novel evodiamine amino derivative as a PI3K/AKT signaling pathway modulator that induces apoptosis in small cell lung cancer cells. Eur. J. Pharmacol..

[20] Proskuriakova E., Khedr A. (2022). Current Targeted Therapy Options in the Treatment of Cholangiocarcinoma: A Literature Review. Cureus.

[21] Trepel J., Mollapour M., Giaccone G., Neckers L. (2010). Targeting the dynamic HSP90 complex in cancer. Nat. Rev. Cancer.

[22] Li Y., Zhang T., Schwartz S.J., Sun D. (2009). New developments in Hsp90 inhibitors as anti-cancer therapeutics: Mechanisms, clinical perspective and more potential. Drug Resist. Updates Rev. Comment. Antimicrob. Anticancer. Chemother..

