# Supplementary material for: Trans-(±)-TTPG-B Attenuates Cell Cycle Progression and Inhibits Cell Proliferation on Cholangiocarcinoma Cells
Source: Molecules. 2023 Oct 30;28(21):7342. doi: 10.3390/molecules28217342 (PMC10650166; doi:10.3390/molecules28217342)
Supplement: Supplementary file 1 [file molecules-28-07342-s001.zip › molecules-2606829-supplementary.pdf]

Table S1. Name and RCSB code of 22 selected targets of (±)-ARC and (±)-TTPG-B.

No.	Protein	PDB name	RMSD (A)
	**Cell signaling**		
1.	ACDY10	4OYA	5.21*
2.	AKT	1GZN	-
3.	CSF1R	4R7H	1.52
4.	EGFR	6DUK	2.03
5.	FLT3	5X02	-
6.	GSK3b	4J71	3.71*
7.	HER2	3PP0	1.73
8.	Hsp90α	3O0I	3.57
9.	Hsp90β	3NMQ	2.81*
10.	JAK1	5HX8	2.05
11.	MDM2	3LBL	2.76
12.	MEK1	2P55	4.71*
13.	mTOR	4JSV	-
14.	NF-kB	1SUC	-
15.	PI3K	3CSF	3.87*
16.	PKC	3IW4	1.87
17.	RIPK	4ITJ	1.39
18.	STAT3	6MBZ	-
	**Cell invasion**		
1.	AKR1B1	4JIR	1.82
2.	VEGFR	4ASE	2.15
	**Cell cycle**		
1.	CDK1	4Y72	3.82
2.	CyclinB1	4Y72	-

The “*” denoted the docked ligand and co-crystallized ligand are different. Therefore, the RMSD was computed based on the best fitting score.
